# Auxin‐Dependent Activation of RHD6‐RSL4 Cascade Promotes Root Hair Growth Under Boron Deficiency in Arabidopsis Primary Root Apices

**DOI:** 10.1111/pce.70601

**Published:** 2026-05-10

**Authors:** Cristina Bahamonde, Clara de la Osa, Jesús Rexach, M. Begoña Herrera‐Rodríguez, Victoria Berdion Gabarain, José Manuel Estevez, M. Teresa Navarro‐Gochicoa, Agustín González‐Fontes, Juan J. Camacho‐Cristóbal

**Affiliations:** ^1^ Departamento de Fisiología, Anatomía y Biología Celular Universidad Pablo de Olavide Sevilla Spain; ^2^ Fundación Instituto Leloir and IIBBA‐CONICET Buenos Aires Argentina; ^3^ ANID ‐ Millennium Science Initiative Program ‐ Millennium Institute for Integrative Biology (iBio) Santiago, Chile and Centro de Biotecnología Vegetal, Facultad de Ciencias de la Vida Universidad Andres Bello Santiago Chile

**Keywords:** Arabidopsis, auxin signaling, boron deficiency, root hair, root plasticity

## Abstract

Plants rapidly adjust their root system architecture to enhance resource uptake and cope with fluctuating soil environments. One such adaptive response is the development of root hairs (RH), which increase the effective root surface area and thereby improve water and nutrient acquisition. Here, we show that Arabidopsis seedlings respond rapidly to boron (B) deficiency by increasing both RH density and length at the root tip. This response is strongly correlated with enhanced auxin signaling in the root apex, as revealed by the auxin‐responsive reporters IAA2::GUS and DR5rev:GFP. Our findings support the idea that B deficiency leads to auxin accumulation in the root tip, likely mediated by enhanced shoot‐to‐root auxin transport. Furthermore, by using pharmacological and genetic approaches, we provide evidence that enhanced auxin signaling via TIR1/AFBs in the RH zone activates the RHD6‐RSL4 cascade to promote RH elongation under B deficiency. In contrast, the increase in RH density under B‐deficient conditions appears to involve additional auxin‐independent regulatory mechanisms.

## Summary statement

Our results provide new insights into how micronutrient availability modulates root hair development and reveal a mechanistic link between boron deficiency, auxin‐mediated signaling, and RHD6‐RSL4‐driven control of root hair elongation.

## 1 Introduction

Boron (B) is a micronutrient necessary for the optimal development of vascular plants, with an essential role in the maintenance and stability of the cell‐wall matrix due to its capacity to cross‐link two rhamnogalacturonan II molecules (Kobayashi et al. 1996; O'Neill et al. 2004; Bolaños et al. 2023). However, B deficiency does not only affect the cell wall but also disturbs a wide range of physiological and metabolic processes that limit yields and quality of crops (Camacho‐Cristóbal et al. 2008; Tanaka and Fujiwara 2008; Brdar‐Jokanović 2020). B deficiency induces significant alterations in the root system architecture of the model plant *Arabidopsis thaliana*, mainly characterized by a reduction in primary root length and an increase in both the number and elongation of root hairs (RH) (Takano et al. 2006; Martín‐Rejano et al. 2011; Camacho‐Cristóbal et al. 2015; González‐Fontes et al. 2016; Herrera‐Rodríguez et al. 2022). Previous studies highlight the existence of a signaling pathway—mediated by the hormones ethylene and auxins, and by reactive oxygen species (ROS)—involved in the rapid inhibition of root cell elongation that occurs when Arabidopsis seedlings are subjected to short‐term B deficiency (Camacho‐Cristóbal et al. 2015; González‐Fontes et al. 2016). By contrast, the molecular mechanisms underlying the induction and elongation of RH in B‐deficient root tip remain poorly characterized. Interestingly, deficiencies in other mineral nutrients, including nitrate, iron, phosphorus, potassium, manganese, and magnesium, similarly promote RH development (Salazar‐Henao et al. 2016; Jia et al. 2023). In many of these cases, ethylene‐, auxin‐, and/or ROS‐mediated signaling pathways have been proposed to regulate RH modifications (Salazar‐Henao et al. 2016; Song et al. 2016; Zhang et al. 2016; Zhu et al. 2016; Mangano et al. 2017, 2018; Bhosale et al. 2018; Liu et al. 2018; Vissenberg et al. 2020).

RH are extensions of epidermal cells that play a crucial role in facilitating nutrient and water uptake, enhancing root anchorage within the soil matrix and mediating responses to both developmental signals and environmental conditions, thereby contributing to overall plant adaptability (Grierson et al. 2014; Lynch 2019; Vissenberg et al. 2020). RH development involves several processes, including cell fate determination, bulge initiation, and hair elongation. These processes have been extensively studied in *Arabidopsis thaliana*, revealing a well‐characterized genetic and molecular pathway (Menand et al. 2007; Yi et al. 2010; Bruex et al. 2012; Balcerowicz et al. 2015; Lin et al. 2015; Shibata and Sugimoto 2019). Briefly, the epidermal cell fate determination into either trichoblast (hair) or atrichoblast (non‐hair) cells depends on positional cues from underlying cortical cells, and is regulated by a complex network involving numerous key transcription factors (TFs) genes. In non‐hair cells, the expression of *GLABLA2* (*GL2*) functions as a negative regulator of root hair formation. In hair cells, the lack of GL2 allows for the activation of *ROOT HAIR DEFECTIVE 6* (*RHD6*) and its homolog *RHD6‐LIKE1* (*RSL1*), leading to the initiation of RH development. Consequently, RHD6 (and RSL1) positively regulates the expression of *ROOT HAIR DEFECTIVE 6‐LIKE2* (*RSL2*) and *ROOT HAIR DEFECTIVE 6‐LIKE4* (*RSL4*), which induce RH elongation. Besides RHD6 and RSLs, TFs from the *ROOT HAIRLESS‐LIKE* (*LRL*) family of *Lotus japonica* have also been implicated in the regulation of RH growth (Karas et al. 2009; Breuninger et al. 2016).

It is well established that phytohormones and environmental conditions modulate the genetic framework underlying RH development (Lopez et al. 2024). Hormones such as auxins, ethylene, and cytokinins directly or indirectly stimulate the RH growth by regulating transcription of the mentioned TFs (Vissenberg et al. 2020; Li et al. 2022). Specifically, auxin is a well‐known phytohormone that strongly promotes root hair growth, and a connection between auxin responses and the RH development network has been established in Arabidopsis (Mangano et al. 2017; Bhosale et al. 2018; Li et al. 2022). The transcriptional auxin response pathway involves the perception of auxin by Transport Inhibitor Response 1/Auxin Signaling F‐Box (TIR1/AFBs) receptors, F‐box proteins of the E3 ubiquitin ligase complex SKP1‐CULLIN1‐Fbox‐TIR1/AFBs (SCF^TIR1/AFBs^), which facilitates the ubiquitination and the subsequent proteasomal degradation of the transcriptional repressor Auxin/Indole‐3‐Acetic Acid (Aux/IAA). As a result, Auxin Response Factor (ARF) proteins are released, enabling them to regulate the expression of auxin‐responsive genes (Mockaitis and Estelle 2008). Previous studies have shown that several ARFs, including ARF5, ARF7, and ARF19, induce *RSL4* expression, supporting the connection between auxin signaling and the RH developmental network (Mangano et al. 2017; Bhosale et al. 2018; Li et al. 2022). Accordingly, Arabidopsis mutants defective in auxin biosynthesis or signaling display hairless or short‐hair phenotypes at the root (Velasquez et al. 2016; Mangano et al. 2017). Interestingly, auxin involvement in the promotion of both RH initiation and growth has been described under low phosphate (Bhosale et al. 2018) or nitrogen (Jia et al. 2023) conditions.

To date, the molecular processes underlying the adaptive RH response to low B availability remain unknown. In this work, we show that B deficiency causes an increase in the length and the density of RH at the root tip. By using different experimental approaches, it was concluded that enhanced auxin signaling in the RH zone activates the RHD6‐RSL4 cascade to promote RH elongation under B deficiency, while increased RH density likely involves additional pathways.

## 2 Materials and Methods

### 2.1 Plant Material and Growth Conditions

Seeds of wild‐type (ecotype Col‐0) and the different Arabidopsis lines described in Supporting Information Table S1 were surface sterilized with 75% (v/v) ethanol for 5 min, then 2% (w/v) hypochlorite solution for 5 min and, finally, washed 6 times with sterile water. Sterilized seeds were stored in sterile water at 4°C for 5 d in darkness to promote synchronized germination. Sterile seeds were sown on square (12 × 12 cm) plates containing 40 mL of sterile solid growth medium and sealed with Parafilm. The growth medium contained half‐strength MS basal salts with 10 μM H_3_BO_3_ (instead of 50 μM H_3_BO_3_), 2 mM MES, and 1% (w/v) sucrose; the pH of the medium was adjusted to 5.7 with KOH and solidified with 1% (w/v) Gelrite (G1101, Duchefa Biochemie). After sowing, the plates were transferred to a growth chamber in a vertical orientation with a light/dark regime of 16/8 h, 25°C/22°C, 75%/75% relative humidity, and a light intensity of 120–150 μmol·m^−2^·s^−1^ of photosynthetically active radiation.

Seedlings were grown in these conditions for 6 d and then used for further analysis.

### 2.2 Root Treatments

At least twenty 6‐d‐old seedlings were carefully transferred to new plates containing solid B‐deficient medium (0.1 μM B) or control medium (10 μM B), typically for 24 h. When indicated, the following reagents were added to the media before solidification: 20 nM indole‐3‐acetic acid (IAA, Sigma‐Aldrich) or 5 µM α‐(phenylethyl‐2‐oxo)‐IAA (PEO‐IAA, a kind gift from Dr. Ken‐ichiro Hayashi).

### 2.3 Measurements of RH Length and Density

After 24 h of treatments, images of the apical segment within 2.5 mm of the primary root were acquired directly from seedlings growing in solid medium using a Leica S8APO Stereozoom microscope equipped with a digital camera (Leica EC3) and driven by analysis software (LAS EZ; Leica, Heerbrugg, Switzerland). The length (LRH) and density (DRH) of RH were measured in each plant by analyzing the digital images using ImageJ software. DRH was calculated considering the length of the RH zone of the apical 2.5 mm segments. Data were exported to an Excel worksheet for final processing.

Values are given as mean ± SD of at least 12–15 separate plants. Each result is representative of at least three independent experiments.

### 2.4 GUS Staining and GFP Signal

For histochemical analysis of GUS reporter enzyme activity, Arabidopsis seedlings were incubated at 37°C in a GUS reaction buffer containing 2 mM 5‐bromo‐4‐chloro‐3‐indolyl‐β‐d‐glucuronide (Sigma‐Aldrich) in 100 mM sodium phosphate (pH 7.0). GUS staining patterns were observed on a Leica S8APO Stereozoom microscope equipped with a digital camera (Leica EC3) driven by analysis software (LAS EZ, Switzerland).

The fluorescence signal of GFP reporter lines was observed with a Zeiss Axioskop fluorescence microscope using an excitation wavelength of 488 nm and detecting the signal between 505 and 530 nm.

GFP and GUS signals were quantified as mean gray value using ImageJ and expressed in AU. For each plant line and for each treatment, at least 10 plants were analyzed in two independent experiments. Representative plant images were chosen for each treatment.

### 2.5 RNA Isolation, cDNA Synthesis, and Quantitative RT‐PCR Analyses

Total RNA was extracted from root apices using Tri‐Reagent RNA/DNA/Protein Isolation Reagent (Molecular Research Center, Cincinnati, OH, USA) and then treated with RNase‐free DNase (Qiagen, Hilden, Germany) according to the manufacturer's instructions. RNA was purified using an RNA Clean & Concentrator column (Zymo Research, Irvine, CA, USA). Two micrograms of DNase‐treated total RNA were used to prepare cDNA by reverse transcription with M‐MLV reverse transcriptase (New England Biolabs Inc., Ipswich, MA, USA) and oligo(dT)18 primers (Bioline, London, UK), according to the manufacturer's protocol. Gene expression was determined by quantitative RT‐PCR (MyiQ real‐time PCR detection system, Bio‐Rad Laboratories Inc., Hercules, CA, USA) using gene‐specific primers (Supporting Information Table S2) and iQ SYBR Green Supermix (Bio‐Rad), following the manufacturer's instructions. The relative expression levels were calculated according to the 2^−ΔΔCT^ method.

The Arabidopsis *EF1 α* (At1g07940), *TON1A* (At3g55000), and *PP2AA3* (At1g13320) amplicons (Supporting Information Table S2) were used as an internal control to normalize all data as described by Vandesompele et al. (2002). The efficiency of the quantitative RT‐PCR reactions was higher than 90%.

Quantitative RT‐PCR analyses were carried out with cDNA synthesized from five biological replicates. Each biological replicate consisted of RNA extracted from a pool of at least 500 root apices per treatment. The measurement for each sample was repeated twice.

### 2.6 Statistical Analyses

Data shown are mean values ± SD. All experiments were repeated at least twice. Data were compared using Student's *t*‐test (pairwise comparisons) or one‐way ANOVA followed by Tukey's post hoc test (multiple comparisons). Significance was set at *p* < 0.05.

## 3 Results

### 3.1 Low B Conditions Induced RH Development in the Root Tip of Wild‐Type Plants

**Figure 1:**
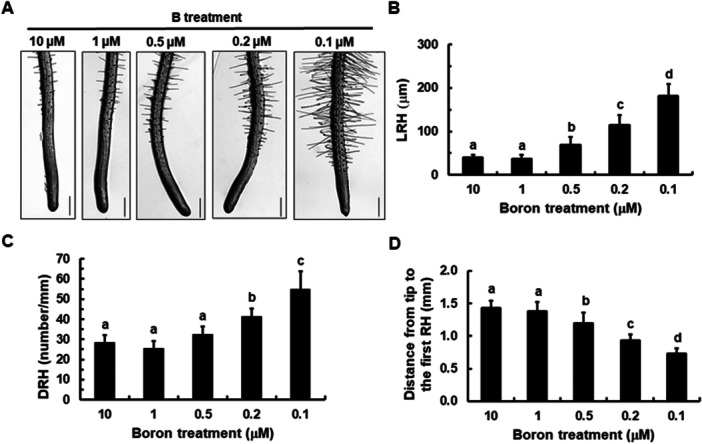
Effect of B deficiency on RH formation in Arabidopsis primary root tip. (A–D) Representative images (A) and quantified RH length (B), density (C), and the distance from the first visible RH to the root tip (D) of wild‐type plants grown under different B supplies. Six‐day‐old seedlings grown on 10 μM B were transferred to culture media containing either 10, 1, 0.5, 0.2, or 0.1 μM B. RHs were imaged and assessed 24 h after transfer. Values are given as mean ± SD of at least 12–15 separate plants for each B treatment. Different letters indicate statistically significant differences at *p* < 0.05 according to one‐way ANOVA and post hoc Tukey test. Scale bars, 300 μm.

First, a time dose response experiment to study the effects of low B treatments on RH development was carried out in wild‐type (WT) seedlings. For this purpose, 6‐d‐old seedlings grown on vertically oriented control medium (10 μM B) were transferred to medium containing different concentrations of B for 24 h. Images of the primary root tip (2.5 mm segment from the root tip) were acquired and examined for RH formation (Figure 1A).

Our external B manipulation elicited robust dose‐dependent RH responses (Figure 1A–D). Within the fixed apical 2.5 mm segment, decreased external B (under 0.5 μM B) is associated with increased LRH (Figure 1B) and DRH (Figure 1C) at 24 h in WT plants. Compared to control (10 μM B), low B supplies at 0.5, 0.2, and 0.1 μM increased LRH by about 100%, 200%, and 400%, respectively (Figure 1B); however, DRH increased by approximately 50% and 100% at 0.2 and 0.1 μM B compared to the control (Figure 1C). Therefore, 0.1 μM B was chosen for B‐deficient treatment in all subsequent experiments since led to a robust response. RH began to develop closer to the root tip in B‐deficient WT plants (0.1 μM B) than in control plants (Figure 1A,D), which is consistent with the rapid inhibition of root cell elongation observed in Arabidopsis seedlings under B deficiency (Camacho‐Cristóbal et al. 2015). Given the rapid inhibition of root cell elongation and the observed proximal shift of the RH zone under B deficiency (Figure 1D), density and length estimates in a fixed apical window may reflect increased occupancy of the mature RH zone under low B; we therefore interpret DRH/LRH as composite measures of RH onset and elongation. Our analyses did not explicitly separate initiation from elongation; future phase‐resolved imaging and reporter mapping will clarify pathway placement by distinguishing bulge formation dynamics from RH growth rates.

### 3.2 Involvement of Auxin in B Deficiency‐Induced RH Development at the Root Tip of Arabidopsis Plants

It is well known that auxin is a key regulator of RH morphogenesis under several stress conditions (Lopez et al. 2024). Therefore, the possible involvement of this hormone in the regulation of low B‐induced RH development was tested. For this purpose, pharmacological and genetic approaches were performed.

**Figure 2:**
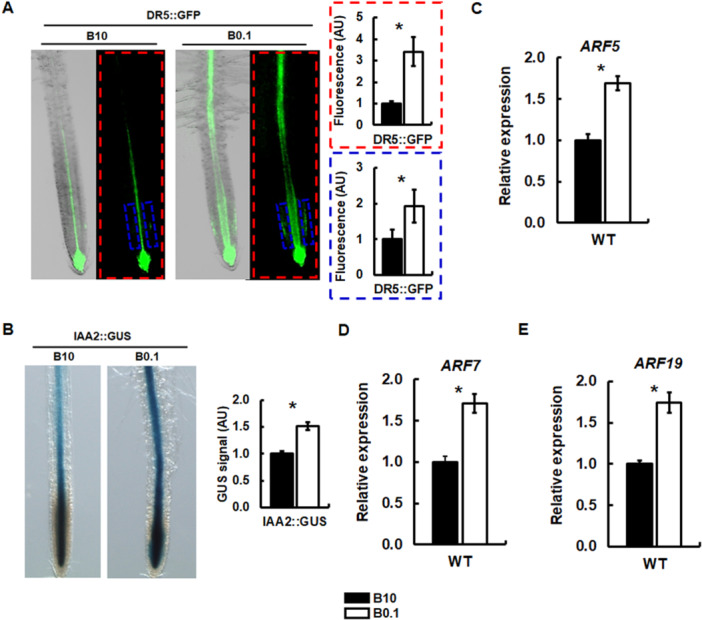
Boron deficiency increases the expression of auxin‐related genes in Arabidopsis primary root tip. (A and B) Representative images (left panel) and relative GFP/GUS signal intensity (right panel) of the auxin‐related marker lines DR5rev::GFP (A) and IAA2::GUS (B) after 24 h of culture under control (10 μM B, B10 and filled bars) or deficiency (0.1 μM B, B0.1 and open bars) conditions. The fluorescence intensity of DR5rev::GFP was measured in the whole apical segments (red boxes) and in the epidermal tissues of the meristematic zone (blue boxes). Values are given as mean ± SD of at least 10 separate plants for each B treatment. Asterisks indicate statistically significant differences between B treatments according to Student's *t*‐test (*p* < 0.05). (C–E) Transcript levels of *ARF5* (C), *ARF7* (D), and *ARF19* (E) genes in the root tip of wild‐type plants after 24 h of culture under control (10 μM B, filled bars) or deficiency (0.1 μM B, open bars) conditions. The results are given as mean ± SD (*n* = 5 separate pools of root apices for each treatment). Asterisks indicate statistically significant differences between B treatments according to Student's *t*‐test (*p* < 0.05).

We first investigated whether the RH response to B deficiency (high increase in LRH and DRH) correlates with an enhancement in endogenous auxin content/signaling. Thus, the auxin related reported lines IAA2::GUS (Swarup et al. 2001) and DR5rev::GFP (Friml et al. 2003) were used. The expression levels of both reported lines clearly increased in the vascular tissues of the root tip upon exposure to B deficiency for 24 h (Figure 2A,B). In addition, an increase in the fluorescence intensity of DR5rev::GFP was observed in the epidermal tissues of the meristematic zone at the root tips after 24 h of B deficiency (Figure 2A, blue boxes). As we did not directly quantify IAA levels in root apices, DR5rev::GFP and IAA2::GUS increases under B deficiency are interpreted as enhanced signaling rather than definitive auxin accumulation. Accordingly, the expression level of several ARF TFs, such as *ARF5*, *ARF7*, and *ARF19*, which coordinate the transcriptional response required for RH elongation (Mangano et al. 2017), increased in root tips after 24 h of B deficiency (Figure 2C–E).

**Figure 3:**
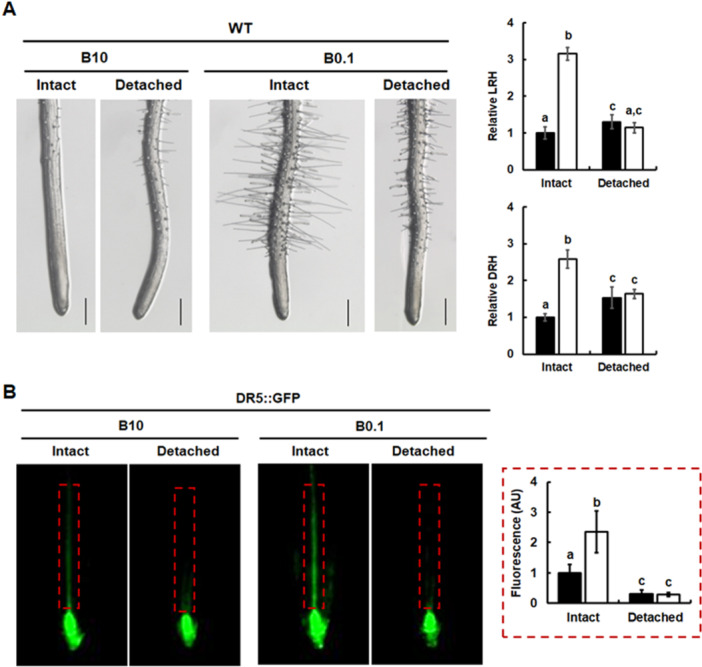
Comparison of RH response and auxin signaling at the tip of detached and intact Arabidopsis primary roots under B deficiency. (A) Representative images (left panel) and quantified RH length and density (right panels) of intact and detached wild‐type primary roots after 24 h of culture under control (10 μM B, B10 and filled bars) or deficiency (0.1 μM B, B0.1 and open bars). Values are expressed relative to control conditions (B10), which was set to 1. Values are given as mean ± SD of at least 12–15 separate plants for each treatment. Different letters indicate statistically significant differences at *p* < 0.05 according to one‐way ANOVA and post hoc Tukey test. Scale bars, 300 μm. (B) Representative images (left panel) and relative GFP signal intensity (right panel) of intact and detached DR5rev::GFP primary roots after 24 h of culture under control (10 μM B, B10 and filled bars) or deficiency (0.1 μM B, B0.1 and open bars) conditions. The fluorescence intensity of DR5rev::GFP was measured in the vascular tissues of the root tip (red boxes). Values are given as mean ± SD of at least 10 separate plants for each B treatment. Different letters indicate statistically significant differences at *p* < 0.05 according to one‐way ANOVA and post hoc Tukey test. [Color figure can be viewed at wileyonlinelibrary.com]

**Figure 4:**
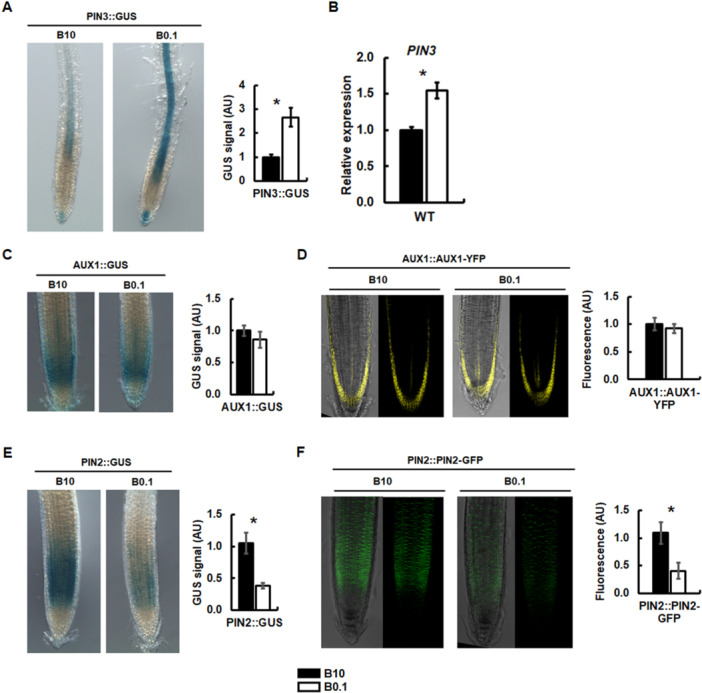
Effect of B deficiency in the expression of auxin transport‐related genes in Arabidopsis primary root tip. (A) Representative images (left panel) and relative GUS signal intensity (right panel) of the PIN3::GUS reporter line after 24 h of culture under control (10 μM B, B10 and filled bars) or deficiency (0.1 μM B, B0.1 and open bars) conditions. (B) Transcript level of *PIN3* gene in the root tip of wild‐type plants after 24 h of culture under control (10 μM B, filled bars) or deficiency (0.1 μM B, open bars) conditions. The results are given as means ± SD (*n* = 5 separate pools of root apices for each treatment). Asterisks indicate statistically significant differences between B treatments according to Student's *t*‐test (*p* < 0.05). (C and D) Representative images (left panel) and relative GUS/YFP signal intensity (right panel) of the AUX1::GUS (C) and AUX1::AUX1‐YFP (D) reporter lines after 24 h of culture under control (10 μM B, B10 and filled bars) or deficiency (0.1 μM B, B0.1 and open bars) conditions. (E and F) Representative images (left panel) and relative GUS/GFP signal intensity (right panel) of the PIN2::GUS (E) and PIN2::PIN2‐GFP (F) reporter lines after 24 h of culture under control (10 μM B, B10 and filled bars) or deficiency (0.1 μM B, B0.1 and open bars) conditions. In (A), (C and D), and (E and F), values are given as mean ± SD of at least 10 separate plants for each B treatment. Asterisks indicate statistically significant differences between B treatments according to Student's *t*‐test (*p* < 0.05). [Color figure can be viewed at wileyonlinelibrary.com]

Experiments were conducted to analyze the effects of B deficiency on auxin biosynthesis‐ and transport‐related genes, which play a critical role in the distribution and accumulation of auxin in root apices. Neither the transcript nor protein levels of TAA1 (Tryptophan Aminotransferase of Arabidopsis 1) and YUC8 (YUCCA8) auxin biosynthesis‐related genes were upregulated in root apices grown under B deficiency (Supporting Information Figure S1). These results would suggest that the B deficiency‐increased auxin signaling in root apices is not based on the local activation of *TAA1*/*YUC8* pathway, contrary to what has been described in the root apex of Arabidopsis plants grown under several abiotic stress conditions (Bhosale et al. 2018; Jia et al. 2023; Berdion Gabarain et al. 2025). To explore the possible involvement of shoot‐derived auxin signaling in the RH response to B deficiency, shoots were immediately removed from 6‐d‐old WT and DR5rev::GFP seedlings that had been transferred to B‐deficient medium, and detached roots were kept in the medium for 24 h (Figure 3). The removal of shoots inhibited the induction of both LRH and DRH in B‐deficient WT plants, although this inhibition was much more pronounced for LRH than for DRH (Figure 3A). Furthermore, shoot removal also led to a strong decrease of the GFP signal in the vascular tissues of B‐deficient DR5rev::GFP root tips (Figure 3B). These results highlight the requirement of shoot‐derived auxin signaling for the RH response (especially the elongation component) to B deficiency. Consistent with this, Tao et al. (2023) reported that B deprivation promotes the polar auxin transport from shoots to roots by upregulating the gene expression levels of the auxin efflux carrier *PIN3*, which results in auxin accumulation in root apices. Accordingly, PIN3::GUS transgenic seedlings show stronger GUS staining in the stele of root tip after 24 h of B‐deficient treatment than in control seedlings (Figure 4A). Also, an increase in *PIN3* transcript levels was observed in B‐deficient roots when compared to control ones (Figure 4B). Although our dataset does not directly test the necessity of shoot‐derived PIN3‐mediated auxin transport under B deficiency, these results are consistent with auxin accumulation via enhanced transport, potentially involving shoot‐derived PIN3 activity.

**Figure 5:**
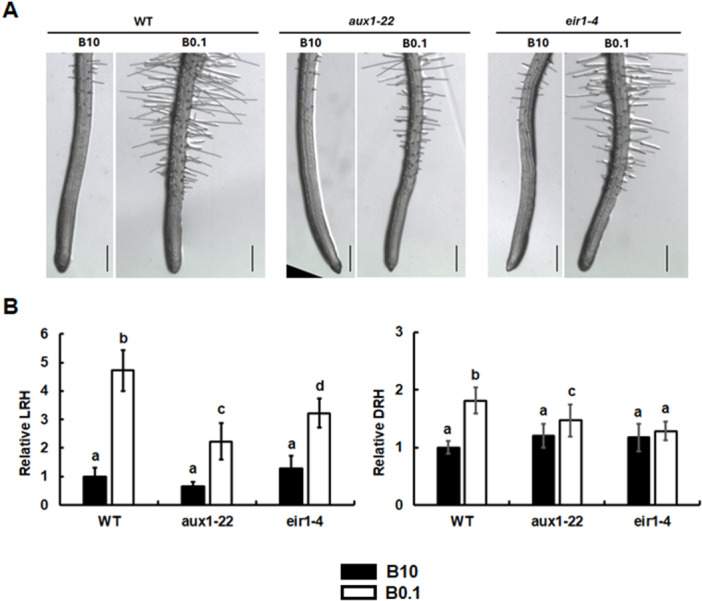
Auxin transport mediated by AUX1 and PIN2 is involved in the RH response to B deficiency in Arabidopsis primary root tip. (A and B) Representative images (A) and quantified RH length and density (B) of wild‐type, *aux1‐22*, and *eir1‐4* (*PIN2*‐related mutant) plants after 24 h of culture under control (10 μM B, B10 and filled bars) or deficiency (0.1 μM B, B0.1 and open bars) conditions. Values are expressed relative to those of the wild‐type under control conditions (B10), which was set to 1. Values are given as mean ± SD of at least 12–15 separate plants for each treatment. Different letters indicate statistically significant differences at *p* < 0.05 according to one‐way ANOVA and post hoc Tukey test. Scale bars, 300 μm.

We next investigated the possible involvement of PIN2 and AUX1, which mediate shootward auxin transport from the root tip to the differentiation zones (Wiśniewska et al. 2006; Swarup and Péret 2012), in the RH response to B deficiency. The expression pattern and signal intensity of AUX1::GUS and AUX1::AUX1‐YFP reporter lines were not affected by B deficiency (Figure 4C,D). By contrast, PIN2‐GUS/GFP levels (expressed as PIN2::GUS and PIN2::PIN2‐GFP) significantly decreased under B deficiency (Figure 4E,F). However, the LRH and, to a lesser extent, DRH of *aux1‐22* and *eir1‐4* (*PIN2*‐related mutant) mutants under B deficiency were significantly lower compared to the WT (Figure 5). Taken together, these results suggest that shootward AUX1‐ and PIN2‐driven auxin transport to the RH zone is important for stimulating the RH response (especially the LRH) to B deficiency. This is consistent with the increase in the fluorescence intensity of DR5rev::GFP observed in the epidermal tissues of the meristematic zone at the root tips under B deficiency (Figure 2A, blue boxes).

**Figure 6:**
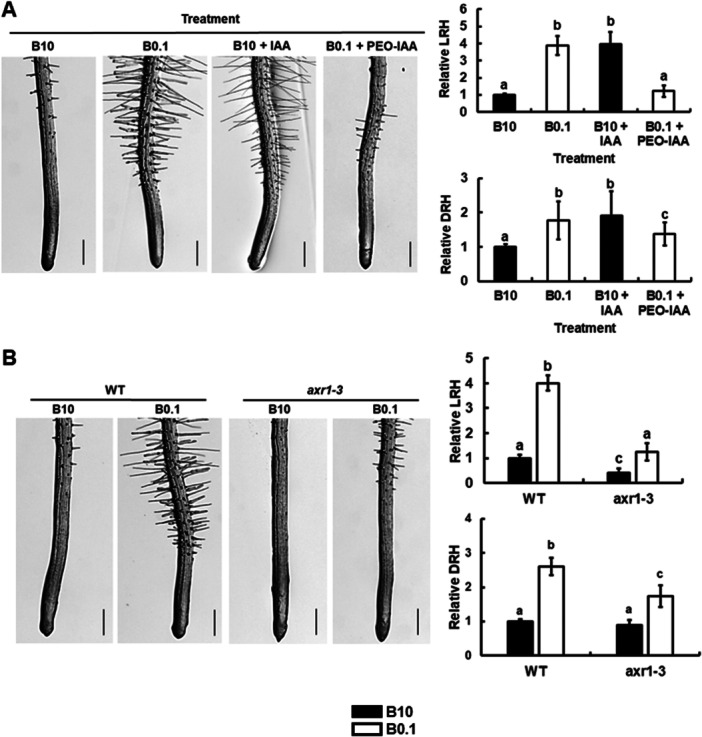
The RH response to B deficiency in Arabidopsis primary root tip requires auxin signaling. (A) Representative images (left panel) and quantified RH length and density (right panels) of wild‐type plants after 24 h of culture under control (10 μM B, B10 and filled bars), control plus 20 nM IAA (B10 + IAA), deficiency (0.1 μM B, B0.1 and open bars), or deficiency plus 5 µM PEO‐IAA (B0.1 + PEO‐IAA) conditions. Values are expressed relative to control conditions (B10), which was set to 1. (B) Representative images (left panel) and quantified RH length and density (right panels) of wild‐type and *axr1‐3* mutant plants after 24 h of culture under control (10 μM B, B10 and filled bars) or deficiency (0.1 μM B, B0.1 and open bars) conditions. Values are expressed relative to that of wild‐type under control conditions (B10), which was set to 1. In (A) and (B), values are given as mean ± SD of at least 12–15 separate plants for each treatment. Different letters indicate statistically significant differences at *p* < 0.05 according to one‐way ANOVA and post hoc Tukey test. Scale bars, 300 μm.

Then, we investigated whether enhanced auxin signaling is required for the induction of RH development by applying the auxin IAA to B‐sufficient plants. Both RH length and density in B‐sufficient WT plants were significantly increased by 20 nM IAA treatment, resembling the phenotype of B‐deficient WT plants (Figure 6A and Supporting Information Figure S2). To further assess the mediation of auxin signaling in the RH response to B deficiency, Arabidopsis seedlings were treated with 5 μM PEO‐IAA (a synthetic antagonist of IAA that binds to TIR1/AFBs auxin receptors) under B deficiency. The induction of LRH in B‐deficient WT plants was abolished in the presence of PEO‐IAA (Figure 6A); however, the presence of PEO‐IAA did not completely inhibit the response of DRH in B‐deficient WT roots (Figure 6A). The involvement of auxin signaling pathway in the response of RH under B deficiency was also tested by using a genetic approach. For this purpose, we used the *axr1‐3* mutant, which is defective in auxin signaling. In fact, AXR1 (AUXIN RESISTANT 1) plays a pivotal role in auxin signaling transduction, as it is required for the accurate function of SCF^TIR1/AFBs^ complex (del Pozo et al. 2002). Accordingly, unlike WT plants, *axr1‐3* mutants were insensitive to IAA exogenous application (Supporting Information Figure S3), and neither LRH nor DHR increased after application of exogenous IAA (Supporting Information Figure S3). Interestingly, the *axr1‐3* mutant displays a decreased LRH compared to WT plants under both B‐sufficient and ‐deficient treatments (Figure 6B). The reduced LRH observed in the *axr1‐3* mutant when compared to WT plants was more evident under B deficiency than in control conditions (Figure 6B), suggesting that the auxin signaling pathway to induce RH growth is more active in B‐deficient roots than in control ones. The *axr1‐3* mutant also showed a decreased DRH compared to WT plants under B deficiency, this effect being less evident than that observed for LRH (Figure 6B). Interestingly, considering that the DRH measurement at 24 h integrates both initiation frequency and early elongation, the persistence of a higher DRH in the presence of PEO‐IAA (Figure 6A) and in the *axr1‐3* (Figure 6B) under low B likely reflects initiation contributions. As time‐resolved initiation assays were not performed, the relative auxin dependence of initiation vs. elongation was inferred from aggregate DRH/LRH metrics. Taking these facts into account, these results indicate that TIR1/AFBs‐dependent auxin perception is required for the elongation component of the B‐deficiency response, while RH initiation may be driven in part by auxin‐independent routes.

### 3.3 B Deficiency Alters the Expression of TFs Involved in RH Development

The expression level of *GL2*, a negative regulator of RH differentiation, was not affected after 24 h of B deficiency when compared to the control treatment (Supporting Information Figure S4A). In addition, the expression pattern of the two well‐characterized atrichoblast marker lines GL2::GUS and GL2::GFP, which reflects *GL2* gene expression in the meristematic and elongation zones of the Arabidopsis root (Masucci et al. 1996; Hung et al. 1998; Lin and Schiefelbein 2001), was not markedly altered under B deficiency (Supporting Information Figure S4C,D). These results indicate that the increase in RH development under B deficiency is not caused by the inhibition of *GL2* gene expression. This hypothesis is also supported by the fact that the loss‐of‐function *gl2‐8* mutant, which showed an increase in root hair density when compared to WT plants (Supporting Information Figure S4B; Shi et al. 2012), exhibited a similar response to B deficiency than WT plants (Supporting Information Figure S4B).

**Figure 7:**
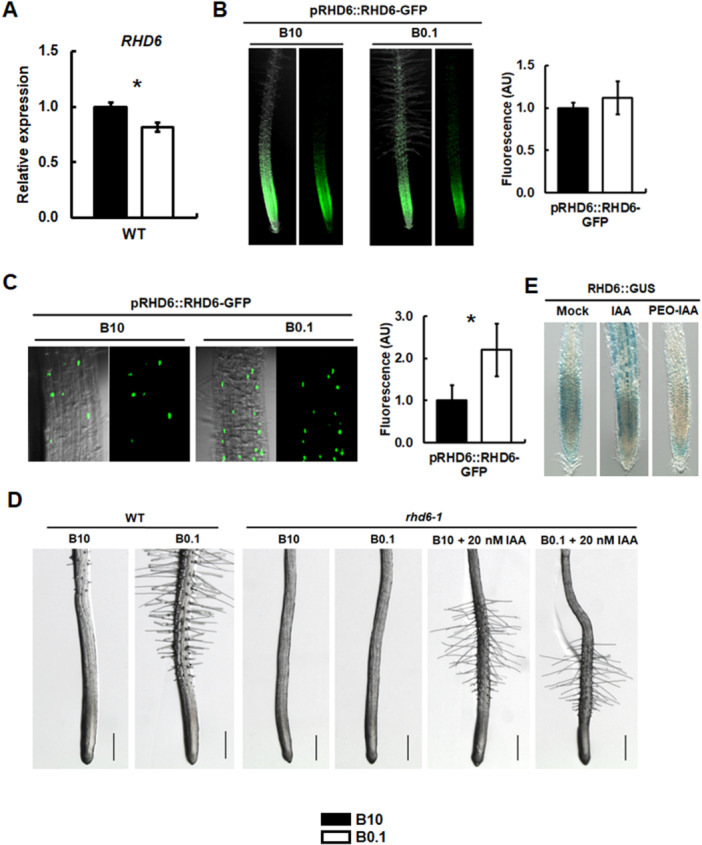
The RH response to B deficiency in Arabidopsis primary root tip requires RHD6. (A) Transcript level of *RHD6* gene in the root tip of wild‐type plants after 24 h of culture under control (10 μM B, filled bars) or deficiency (0.1 μM B, open bars) conditions. The results are given as mean ± SD (*n* = 5 separate pools of root apices for each treatment). Asterisks indicate statistically significant differences between B treatments according to Student's *t*‐test (*p* < 0.05). (B and C) Representative images (left panel) and relative GFP signal intensity (right panel) line in the root tip (B) and differentiation zone (C) of the pRHD6::RHD6‐GFP reporter line after 24 h of culture under control (10 μM B, B10 and filled bars) or deficiency (0.1 μM B, B0.1 and open bars) conditions. Values are given as mean ± SD of at least 10 separate plants for each B treatment. Asterisks indicate statistically significant differences between B treatments according to Student's *t*‐test (*p* < 0.05). (D) Representative images of wild‐type and *rhd6‐1* mutant plants after 24 h of culture under control (10 μM B, B10) or deficiency (0.1 μM B, B0.1) conditions. Scale bars, 300 μm. (E) Representative images of RHD6::GUS line in response to IAA supply or in presence of the auxin‐signaling inhibitor PEO‐IAA. [Color figure can be viewed at wileyonlinelibrary.com]

The expression level of *RHD6*, which promotes the initiation of RH and activates a transcriptional cascade involving RSL2 and RSL4 to stimulate their elongation (Menand et al. 2007; Yi et al. 2010; Datta et al. 2015), slightly decreased in the root tip after 24 h of B deficiency when compared to control treatment (Figure 7A). The accumulation of the RHD6 protein was studied by using a pRHD6::RHD6‐GFP translational fusion line (an early RH development marker line; Borassi et al. 2020) (Figure 7B,C). The RHD6‐GFP signal was mainly accumulated in the meristematic zone but decreased throughout the elongation/differentiation/maturation zones of the root tip (Figure 7B) (Singh et al. 2008; Borassi et al. 2020), which confirms that RHD6 acts at an early stage of RH initiation. Interestingly, despite the slight decrease in *RHD6* expression level observed in the root tip under B deficiency, the RHD6‐GFP signal was significantly enhanced in the differentiation zone of Arabidopsis root tip exposed to 24 h of B deficiency (Figure 7C). In addition, the RH response to B deficiency was fully abolished in the loss‐of‐function *rhd6‐1* mutant (Figure 7D), indicating that RHD6 is required for B deficiency‐induced RH formation. We then tested the possible interaction between auxin signaling and *RHD6* expression by using the transcriptional reporter RHD6::GUS. Results showed that IAA supply significantly increased the activity of *RHD6* promoter in the RH zone, while PEO‐IAA supply largely suppressed it (Figure 7E). In addition, the RH growth of the *rhd6‐1* mutant was restored by exogenous supply of 20 nM IAA in both B‐sufficient and ‐deficient conditions (Figure 7D). These results suggest that auxin can modulate *RHD6* expression and that RHD6 functions upstream of auxin signaling to regulate RH growth.

**Figure 8:**
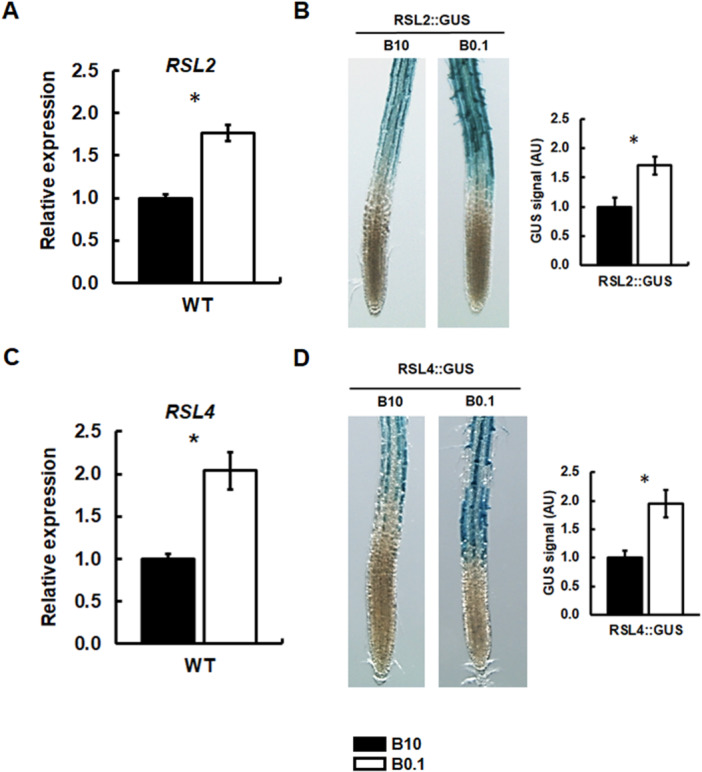
The expression of root hair‐related TFs *RSL2* and *RSL4* in Arabidopsis primary root tip increases under B deficiency. (A and C) Transcript levels of *RSL2* (A) and *RSL4* (C) genes in the root tip of wild‐type plants after 24 h of culture under control (10 μM B, filled bars) or deficiency (0.1 μM B, open bars) conditions. The results are given as mean ± SD (*n* = 5 separate pools of root apices for each treatment). Asterisks indicate statistically significant differences between B treatments according to Student's *t*‐test (*p* < 0.05). (B and D) Representative images (left panel) and relative GUS signal intensity (right panel) of the RSL2::GUS (B) and RSL4::GUS (D) reporter lines after 24 h of culture under control (10 μM B, B10 and filled bars) or deficiency (0.1 μM B, B0.1 and open bars) conditions. Values are given as mean ± SD of at least 10 separate plants for each B treatment. Asterisks indicate statistically significant differences between B treatments according to Student's *t*‐test (*p* < 0.05). [Color figure can be viewed at wileyonlinelibrary.com]

**Figure 9:**
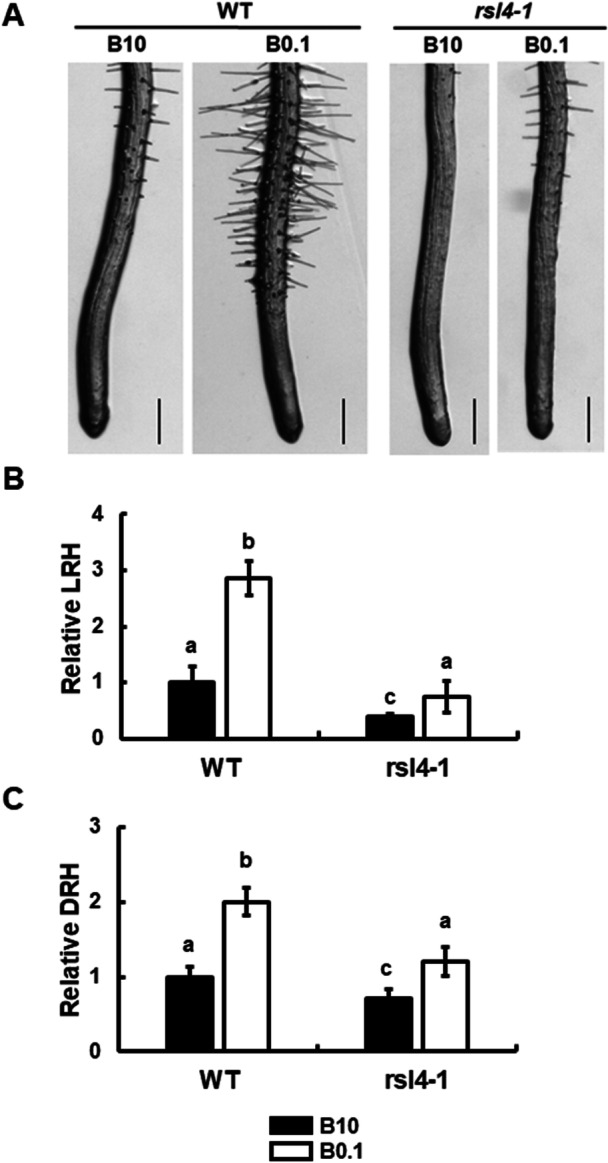
The RH response to B deficiency in Arabidopsis primary root tip requires RSL4. (A–C) Representative images (A) and quantified RH length (B) and density (C) of wild‐type plants and *rsl4‐1* mutant plants after 24 h of culture under control (10 μM B, B10 and filled bars) or deficiency (0.1 μM B, B0.1 and open bars) conditions. Values are expressed relative to those of the wild‐type under control conditions (B10), which was set to 1, and given as the mean ± SD of at least 12–15 separate plants for each treatment. Different letters indicate statistically significant differences at *p* < 0.05 according to one‐way ANOVA and post hoc Tukey test. Scale bars, 300 μm.

**Figure 10:**
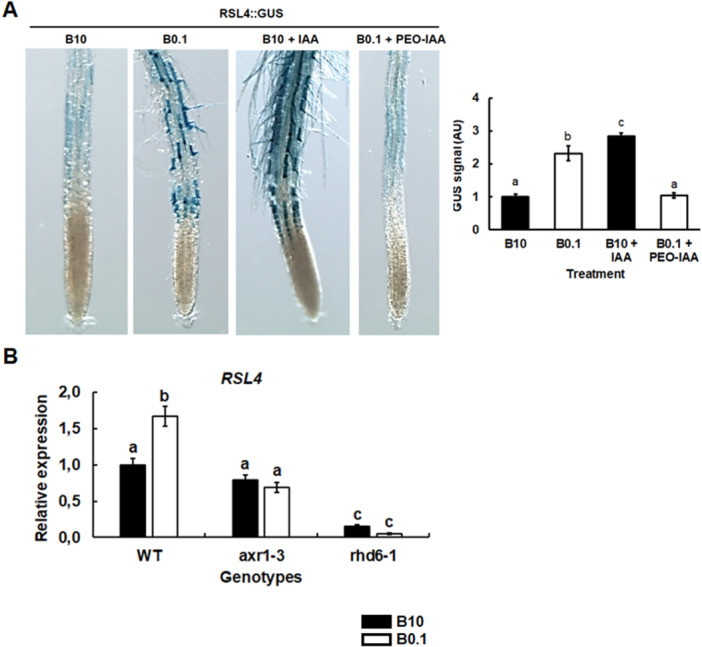
The induction of *RSL4* expression in the root tip of B‐deficient plants requires auxin signaling. (A) Representative images (left panel) and relative GUS signal intensity (right panels) of RSL4::GUS reporter line after 24 h of culture under control (10 μM B, B10 and filled bars), deficiency (0.1 μM B, B0.1 and open bars), control plus 20 nM IAA (B10 + IAA), or deficiency plus 5 µM PEO‐IAA (B0.1 + PEO‐IAA) conditions. Values are given as the mean ± SD of at least 10 separate plants for each B treatment. Different letters indicate statistically significant differences at *p* < 0.05 according to one‐way ANOVA and post hoc Tukey test. (B) Transcript level of *RSL4* in the root tip of wild‐type, *axr1‐3*, and *rhd6‐1* plants after 24 h of culture under control (10 μM B, filled bars) or deficiency (0.1 μM B, open bars) conditions. The results are given as means ± SD (*n* = 5 separate pools of root apices for each treatment). Different letters indicate statistically significant differences at *p* < 0.05 according to one‐way ANOVA and post hoc Tukey test. [Color figure can be viewed at wileyonlinelibrary.com]

After initiation, polar growth of RH is regulated by RSL2 and RSL4 (Yi et al. 2010; Datta et al. 2015), whose expressions notably increased in roots after 24 h of B deficiency (Figure 8A,C). The expression pattern of both TFs was also examined by using the marker lines RSL2::GUS and RSL4::GUS (Figure 8B,D). In agreement with qRT‐PCR results, GUS staining revealed that the expression of both *RSL2* and *RSL4* was induced in roots under B deficiency, especially in the maturation zone (Figure 8B,D). To further explore the possible role of RSL2 and RSL4 in the induction of RH development under B deficiency, the response of the loss‐of‐function *rsl2‐1*, *rsl4‐1*, and *rsl2‐1/rsl4‐1* mutants to B deficiency was also studied. In our experimental conditions, RH response in the *rsl2‐1* mutant was like that observed in WT plants under both control and B‐deficient treatments (Supporting Information Figure S5). Therefore, despite the observed increase in its expression level under B deficiency (Figure 8A), our results suggest that RSL2 does not play a major role in the RH response to B deficiency. This agrees with the well‐known fact that RSL2 functions redundantly with RSL4, having a minor role in RH regulation (Yi et al. 2010). Conversely, the *rsl4‐1* mutant had decreased LRH and DRH compared to WT plants under B‐sufficient treatment (Figure 9), indicating an essential role of RSL4 in RH regulation (Yi et al. 2010). In addition, as in *axr1‐3* (Figure 6B), the response of both LRH and DRH to B deficiency was disrupted in the *rsl4‐1* mutant when compared to WT plants, this effect being more pronounced for LRH than DRH (Figure 9). These results suggest that RSL4 strongly contributes to RH elongation under low B. However, the *rsl4‐1* mutant showed a slight increase in LRH and DRH under B deficiency, which could be explained by the action of RSL2 in the absence of RSL4. This agrees with the phenotype shown by the *rsl2‐1/rsl4‐1* double mutant under B‐deficient conditions, which is not capable of developing visible RH (Supporting Information Figure S6). Interestingly, the double mutant was able to develop RH bulges in the maturation zone under B deficiency (Supporting Information Figure S6), probably through the mediation of RHD6. This hypothesis is supported by the fact that the loss‐of‐function *rhd6‐1* mutant was not capable of developing RH bulges under B deficiency (Figure 7D). On the other hand, as expected, the expression levels of *RSL4* in the root tips of the *rhd6‐1* mutant were very low and, in contrast to WT plants, did not increase under B deficiency (Figure 10B). This result suggests that RHD6 directly activates RSL4 transcription (Yi et al. 2010).

Finally, we also tested the effect of IAA and PEO‐IAA supplies on the RH response of *rsl4‐1* mutant under B‐sufficient and ‐deficient conditions, respectively (Supporting Information Figure S7). IAA stimulated RH growth under B‐sufficient, whereas PEO‐IAA abolished the slight RH response of the *rsl4‐1* null mutant under B deficiency (Supporting Information Figure S7). These results suggest that, in the absence of RSL4, the increase in the auxin signaling pathway can stimulate RH response by the action of RSL2, which would explain the weak RH response to B deficiency shown by the *rsl4‐1* mutant (Figure 9A and Supporting Information Figure S7). Conversely, exogenous IAA supply failed to stimulate RH growth in the *rsl2‐1/rsl4‐1* double mutant (Supporting Information Figure S7), suggesting that RSL4 (and RSL2) acts downstream of auxin signaling to regulate RH growth. Indeed, a previous report suggests that endogenous auxin activates several ARFs to up‐regulate the expression of *RSL4*, linking auxin stimulation to *RSL4* expression at the molecular level (Mangano et al. 2017). In agreement, as above mentioned, the expression levels of *ARF5*, *ARF7*, and *ARF19* increased in root tips after 24 h of B deficiency (Figure 2C–E). To further analyze the interactions between auxin and *RSL4* expression, we studied the effect of auxin or PEO‐IAA treatment on the expression pattern of *RSL4* by using the RSL4::GUS marker line (Figure 10A). Auxin treatment caused a high increase in the expression of *RSL4* under control conditions; however, the induction of *RSL4* expression in B‐deficient plants was inhibited in the presence of PEO‐IAA (Figure 10A). Then, we tested the expression levels of *RSL4* in the root tips of *axr1‐3* mutant (Figure 10B). The mutant *axr1‐3* failed in enhancing *RSL4* mRNA levels under B deficiency (Figure 10B), which correlated with its lower RH development in comparison to WT plants under this abiotic stress (Figure 6B). Therefore, these results show that the induction of *RSL4* expression in the root tip of B‐deficient plants requires auxin signaling and the expression levels of *ARF5*, *ARF7*, and *ARF19* increased in root tips after 24 h of B deficiency. Although we did not assess ARF occupancy or auxin response elements function in the *RSL4* promoter under B deficiency, these data would indicate that auxin signaling via TIR1/AFBs modulates *RSL4* expression under B deficiency.

## 4 Discussion

Plants can quickly modify their root systems to optimize resource acquisition and adapt to changing soil conditions. These modifications include the development of root hairs (RH), which increase the active root surface and facilitate nutrients and water absorption (Rongsawat et al. 2021). Numerous studies have shown that deficiencies in essential nutrients, such as nitrate, iron, phosphorus, potassium, manganese, and magnesium strongly promote RH morphogenesis (Salazar‐Henao et al. 2016; Jia et al. 2023). Although B deficiency has also been reported to cause an increase in both the number and elongation of RH in Arabidopsis roots (Takano et al. 2006; Martín‐Rejano et al. 2011; Camacho‐Cristóbal et al. 2015; González‐Fontes et al. 2016), the molecular mechanisms underlying this response remain poorly characterized. In this study we demonstrate that auxin plays a key role in the induction of RH development in Arabidopsis primary root apices.

Our results demonstrate that *Arabidopsis thaliana* seedlings respond rapidly to B deficiency by increasing both the initiation and elongation of RH in the root tip (Figure 1), this effect being strongly correlated with increased auxin signaling in the root tip. In fact, using DR5‐ and IAA2‐based auxin reporters we observed an increased auxin signaling in the root tip (especially in the vascular tissues of meristematic, elongation and maturation zones and in the epidermal tissues of the meristematic zone) upon exposure to B deficiency (Figure 2A,B; Camacho‐Cristóbal et al. 2015; Tao et al. 2023). It is widely accepted that auxin accumulation within root tip depends on two processes: (i) the transport of shoot‐derived auxin supply through cells in the central cylinder (Muday and DeLong 2001; Friml 2003) and/or (ii) the root‐specific auxin biosynthesis (Ljung et al. 2005; Lv et al. 2019). Previous studies have shown that increased auxin content in the root tips of Arabidopsis exposed to abiotic stress, such as aluminum toxicity, deficiencies in nitrogen or phosphate, or low temperature conditions, is due to a local increase in the expression of genes related to auxin biosynthesis like *TAA1* and/or *YUC8* (Liu et al. 2016; Bhosale et al. 2018; Li et al. 2021; Jia et al. 2023; Berdion Gabarain et al. 2025), among others. However, Tao et al. (2023) reported that B deprivation upregulates the expression of auxin biosynthesis‐related genes in shoots but does not alter their expression in roots, and they concluded that the highest auxin accumulation in the root tip of B‐deficient plants depends on shoot‐derived auxin supply mediated by PIN3. Together with increased *PIN3* expression (Figure 4A,B; Tao et al. 2023) and the absence of *TAA1/YUC8* induction in roots (Supporting Information Figure S1), the concordant DR5rev::GFP and IAA2::GUS signals (Figure 2A,B) support transport‐mediated enhancement of auxin signaling in the root tip under B deficiency. Accordingly, the removal of shoots—which blocks the shoot‐to‐root auxin transport—inhibited the increase of DR5rev::GFP‐derived fluorescence in the vascular tissues of root tips under B deficiency (Figure 3B). This finding, together with the fact that shoot removal also inhibited the RH response to B deficiency in WT plants (in particular the LRH response, Figure 3A), indicates that shoot‐derived auxin is required for both the increase in auxin signaling and the elongation component of the RH response to B deficiency.

It is well known that the auxin carriers AUX1 and PIN2, which facilitate auxin transport toward the elongation and differentiation zones in the root tips, contribute to RH elongation under nitrogen or phosphate deficiencies (Bhosale et al. 2018; Lin et al. 2020; Jia et al. 2023). Even though we did not observe an increase in the expression levels or protein contents of these auxin carriers (Figure 4C–F), the LRH response of *aux1‐22* and *eir1‐4* (*PIN2*‐related mutant) under B deficiency was significantly lower than in WT plants (Figure 5). These results, together with the increased DR5rev::GFP signal in the epidermal tissues of the meristematic zone under low B conditions (Figure 2A, blue boxes), would indicate that AUX1 and PIN2 may facilitate the auxin mobility toward the root RH zone to promote the LRH response under B deficiency.

The increased auxin signaling in the maturation zone of the root tip under B deficiency could explain the promotion of RH response under this stress. Consistent with this, the application of exogenous IAA phenocopied the effects of B deficiency (Figure 6A and Supporting Information Figure S2), while both pharmacological (PEO‐IAA) and genetic (*axr1‐3*) disruption of the auxin signaling pathway dramatically reduced the LRH response under low B conditions (Figure 6A,B). This confirms that the canonical auxin signaling pathway mediated by TIR1/AFBs complex is essential for the LRH response during B deficiency. Accordingly, there is evidence showing the involvement of the auxin signaling pathway in the RH growth of Arabidopsis plants subjected to abiotic stresses such phosphate or nitrogen deficiencies and low temperature (Mangano et al. 2017; Bhosale et al. 2018; Jia et al. 2023; Lopez et al. 2024; Berdion Gabarain et al. 2025). Despite the above, neither the presence of PEO‐IAA nor the genetic disruption of the auxin signaling pathway (*axr1‐3*) completely inhibited the DRH response to B deficiency (Figure 6A,B), which suggests the existence of auxin‐independent or partially redundant pathways involved in RH initiation under B deficiency. In this regard, the promotion of ethylene signaling observed in Arabidopsis roots under B deficiency (Martín‐Rejano et al. 2011; Camacho‐Cristóbal et al. 2015; González‐Fontes et al. 2016) would converge on the EIN3 TF, which physically interacts with RHD6 to promote RH initiation (Feng et al. 2017).

The TFs involved in the first stages of RH development (epidermal cell fate determination, bulge initiation and root hair elongation) are well characterized in Arabidopsis (Vissenberg et al. 2020). Briefly, previous studies have demonstrated that the RH cell fate depends on the absence of an inhibitory pathway that prevents differentiation into a hair cell. One of the key components in this network is the GL2 homeodomain TF, which in turn inhibits the expression of the basic helix‐loop‐helix TFs like *RHD6* (and its partially redundant homolog *RSL1*), *RSL2*, and *RSL4* required for the initiation and elongation of RH (Balcerowicz et al. 2015; Yasin et al. 2024). Interestingly, the fact that *GL2* expression and GL2::GUS/GFP signals remained unchanged in low B conditions (Supporting Information Figure S4), and that the *gl2‐8* mutant responded similarly to WT (Supporting Information Figure S4), suggest that the RH response under B deficiency is not due to alterations in epidermal cell fate, but rather to enhanced growth of hair cells already determined by positional cues. Thus, B deficiency may specifically activate the RH developmental program downstream of cell fate determination.

Previous studies have reported the requirement of RHD6 for RH development under low phosphate (Feng et al. 2017; Bhosale et al. 2018), low nitrogen (Jia et al. 2023), and low‐temperature conditions (Moison et al. 2021; Pacheco et al. 2021). Our findings reveal that RHD6 also plays a critical role in mediating the RH response to low B, as RHD6 protein accumulation significantly increased in the differentiation zone of Arabidopsis root tip upon B deficiency (Figure 7C), and the RH response to B deficiency was completely abolished in the *rhd6‐1* mutant (Figure 7D). Although our findings do not provide direct evidence that the enhanced RHD6 accumulation in the RH zone under B deficiency is driven by auxin signaling, they are consistent with an auxin‐dependent regulation of *RHD6* expression. Indeed, *RHD6* promoter activity was significantly enhanced in the RH zone by exogenous IAA but decreased when auxin signaling was inhibited (Figure 7E; Jia et al. 2023). These results suggest that the enhanced auxin signaling triggered by B deficiency could promote RH response through RHD6‐dependent pathway. Despite this, previous findings showed that exogenous auxin supply could rescue RH elongation of *rhd6* mutants under non‐stressed conditions (Yi et al. 2010; Bruex et al. 2012), indicating that auxin can promote RH growth through an RHD6‐independent pathway. In the present work, exogenous IAA supply was capable of rescuing RH growth in *rhd6‐1* mutants under both B‐sufficient and ‐deficient conditions (Figure 7D). However, the fact that *rhd6‐1* mutant did not form RH in the absence of exogenous IAA either in B‐sufficient or ‐deficient conditions (Figure 7D) would indicate that this RHD6‐independent pathway to stimulate RH growth is not active in our experimental conditions (possibly because endogenous auxin levels are insufficient to activate this pathway).

It is well known that RHD6 is required for the initiation of RH and for stimulating RH elongation through activation of RSL2 and RSL4 (Menand et al. 2007; Yi et al. 2010; Datta et al. 2015). Interestingly, although both the transcript level and spatial expression of *RSL4* and *RSL2* markedly increased under B deficiency (Figure 8), the RH phenotypes of the *rsl2‐1* (Supporting Information Figure S5), *rsl4‐1* (Figure 9), and *rsl2‐1/rsl4‐1* (Supporting Information Figure S6) mutants under B deficiency indicate that RSL4 is a major downstream effector of RH elongation under low B, with RSL2 acting redundantly. In addition, the capacity of the *rsl2‐1*/*rsl4‐1* double mutant to only form bulges in the RH zone under B deficiency (Supporting Information Figure S6) supports the model in which RHD6 is required for initiation, but elongation primarily depends on RSL4. Moreover, the expression levels of *RSL4* in the root tips of the *rhd6‐1* mutant were very low and, in contrast to WT plants, did not increase under B deficiency (Figure 10B), which suggests that RHD6 directly activates *RSL4* transcription (Yi et al. 2010). The activation of this RHD6‐RSL4 cascade to increase the RH growth has also been described under phosphate deficiency (Yi et al. 2010; Bhosale et al. 2018) and low temperature conditions (Pacheco et al. 2021; Berdion Gabarain et al. 2025; Urzúa Lehuedé et al. 2025).

Our data suggest that TIR1/AFB‐RSL4 signaling primarily regulates elongation, whereas increased RH density under B deficiency likely involves additional pathways. This is supported by the following evidence: (i) exogenous IAA treatment increased RSL4::GUS activity under control conditions, while PEO‐IAA repressed *RSL4* expression under B deficiency (Figure 10A), (ii) the auxin‐insensitive *axr1‐3* mutant failed to induce *RSL4* expression under B deficiency (Figure 10B), correlating with its attenuated RH phenotype (Figure 6B), and (iii) the *rsl2‐1/rsl4‐1* double mutant failed to form RH after exogenous IAA supply (Supporting Information Figure S7). It is well known that downstream of TIR1/AFBs‐mediated auxin perception, ARF TFs coordinate the transcriptional response required for RH elongation; in fact, previous works have shown that ARF5, ARF7, and ARF19 directly regulate *RSL4* expression in response to auxin (Mangano et al. 2017; Bhosale et al. 2018; Li et al. 2022). The observed upregulation of these ARFs under B deficiency (Figure 2C,D) supports a model where B deficiency enhances auxin signaling in the root tip, activating ARF TFs that directly induce *RSL4* expression, leading to RH elongation.

**Figure 11:**
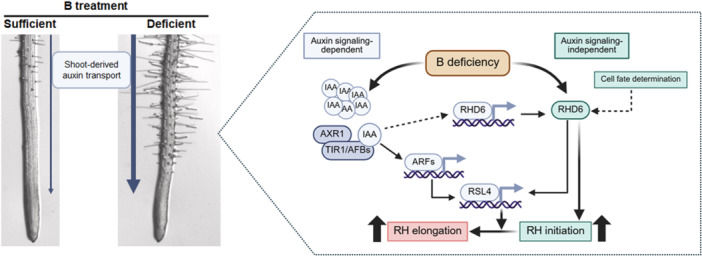
Schematic illustration showing the mechanisms underlying the increased RH initiation and elongation observed under B deficiency in Arabidopsis primary root tip. This model proposes that B deficiency leads to an enhanced auxin signaling in the root tip, which is dependent on shoot‐derived auxin supply. Although we cannot directly link the increased RHD6 accumulation in the RH zone observed under B deficiency to auxin signaling, our findings are consistent with auxin‐dependent upregulation of RHD6 expression, potentially acting upstream of RSL4‐mediated RH elongation. Additionally, we provide evidence that increased auxin signaling, perceived by TIR1/AFB receptors and transduced by specific ARFs (ARF5, ARF7, and ARF19), activates the expression of RSL4 to promote RH elongation under B‐deficient conditions. In summary, the activation of the auxin‐dependent pathway under B deficiency would trigger the downstream RHD6‐RSL4 cascade that mainly underlies the enhanced RH elongation. However, the increase in RH initiation mediated by RHD6 under B‐deficient conditions likely involves additional regulatory inputs that act independently of auxin. [Color figure can be viewed at wileyonlinelibrary.com]

In conclusion, our results support a model (Figure 11) in which B deficiency triggers polar auxin transport from shoot to the primary root apex, followed by shootward auxin redistribution, leading to enhanced auxin signaling in the RH zone. This activates the RHD6–RSL4 cascade, which predominantly drives root hair elongation, whereas root hair initiation likely requires additional regulatory inputs. This response may represent an adaptive mechanism that enhances nutrient acquisition under B‐limiting conditions, increasing the absorptive surface area of the root.

## Conflicts of Interest

The authors declare no conflicts of interest.

## Supporting information

Supporting File:

## Data Availability

The data that support the findings of this study are available from the corresponding author upon reasonable request.
