# Supplementary material for: Auxin‐Dependent Activation of RHD6‐RSL4 Cascade Promotes Root Hair Growth Under Boron Deficiency in Arabidopsis Primary Root Apices
Source: Plant Cell Environ. 2026 May 10;49(8):6081–99. doi: 10.1111/pce.70601 (PMC13353740; doi:10.1111/pce.70601)
Supplement: Supplementary file 1 — Supporting File: [file PCE-49-6081-s001.docx]

**Table S1. Plants used in this work.**

| Lines | Description | Source |
| --- | --- | --- |
| Columbia (Col-0) | Wild type (WT) | Nottingham Arabidopsis Stock Centre (NASC) |
| IAA2::GUS | WT (Col-0) plants expressing GUS under the control of the auxin-inducible *IAA2* promoter | Swarup et al., 2001 |
| DR5rev::GFP | WT (Col-0) plants expressing GFP under the control of an auxin-inducible promoter | Friml et al., 2003 |
| PIN3::GUS | WT (Col-0) plants expressing GUS under the control of *PIN3* promoter | Friml et al., 2002 |
| TAA1::TAA1-GFP | WT (Col-0) plants expressing TAA1-GFP fusion protein under the control of *TAA1* promoter | Yang et al., 2014 |
| YUC8::YUC8-GFP | WT (Col-0) plants expressing YUC8-GFP fusion protein under the control of *YUC8* promoter | Jia et al., 2023 |
| AUX1::GUS | WT (Col-0) plants expressing GUS under the control of *AUX1* promoter | Marchant et al., 1999 |
| AUX1::AUX1-YFP | WT (Col-0) plants expressing AUX1-YFP fusion protein under the control of *AUX1* promoter | Swarup et al., 2004 |
| PIN2::GUS | WT (Col-0) plants expressing GUS under the control of *PIN2* promoter | Friml et al., 2003 |
| PIN2::PIN2-GFP | WT (Col-0) plants expressing PIN2- GFP fusion protein under the control of *PIN2* promoter | Blilou et al., 2005 |
| *aux1-22* | Homozygous *AUX1* mutant | NASC N9585 (Swarup et al., 2005) |
| *eir1-4* | Homozygous *PIN2* mutant | Xu et al., 2012 |
| *axr1-3* | Homozygous *AXR1* mutant | NASC N3075 (Lincoln et al., 1990) |
| GL2::GUS | WT (Col-0) plants expressing GUS under the control of *GL2* promoter | Masucci and Schiefelbein, 1994 |
| GL2::GFP | WT (Col-0) plants expressing GFP under the control of *GL2* promoter | Lin and Schiefelbein, 2001 |
| *gl2-8* | Homozygous *GL2* mutant | NASC N2106731 (Shi et al., 2012) |
| RHD6::RHD6-GFP | WT (Col-0) plants expressing RHD6-GFP fusion protein under the control of *RHD6* promoter | Borassi et al., 2020 |
| RHD6::GUS | WT (Col-0) plants expressing GUS under the control of *RHD6* promoter | Lin et al., 2015 |
| *rhd6-1* | Homozygous *RHD6* mutant | NASC N6347 (Menand et al., 2007) |
| RSL2::GUS | WT (Col-0) plants expressing GUS under the control of *RSL2* promoter | Lin et al., 2015 |
| RSL4::GUS | WT (Col-0) plants expressing GUS under the control of *RSL4* promoter | Yi et al., 2010 |
| *rsl2-1* | Homozygous *RSL2* mutant | Yi et al., 2010 |
| *rsl4-1* | Homozygous *RSL4* mutant | Yi et al., 2010 |
| *rsl2-1/rsl4-1* | Homozygous double mutant *RSL2* and *RSL4* | Yi et al., 2010 |

Blilou I, Xu J, Wildwater M, Willemsen V, Paponov I, Friml J, Heidstra R, Aida M, Palme K, Scheres B, 2005. The PIN auxin efflux facilitator network controls growth and patterning in Arabidopsis roots. Nature 433: 39-44. doi: 10.1038/nature03184.

Borassi C, Gloazzo Dorosz J, Ricardi MM, Carignani Sardoy M, Pol Fachin L, Marzol E, Mangano S, Rodríguez Garcia DR, Martínez Pacheco J, Rondón Guerrero YDC, Velasquez SM, Villavicencio B, Ciancia M, Seifert G, Verli H, Estevez JM, 2020. A cell surface arabinogalactan-peptide influences root hair cell fate. New Phytol 227: 732-743. doi: 10.1111/nph.16487.

Friml J, Vieten A, Sauer M, Weijers D, Schwarz H, Hamann T, Offringa R, Jürgens G, 2003. Efflux-dependent auxin gradients establish the apical-basal axis of Arabidopsis. Nature 426: 147-53. doi: 10.1038/nature02085.

Friml J, Wiśniewska J, Benková E, Mendgen K, Palme K, 2002. Lateral relocation of auxin efflux regulator PIN3 mediates tropism in Arabidopsis. Nature 415: 806-9. doi: 10.1038/415806a.

Jia Z, Giehl RFH, Hartmann A, Estevez JM, Bennett MJ, von Wirén N, 2023. A spatially concerted epidermal auxin signaling framework steers the root hair foraging response under low nitrogen. Curr Biol 33: 3926-3941.e5. doi: 10.1016/j.cub.2023.08.040.

Lin Q, Ohashi Y, Kato M, Tsuge T, Gu H, Qu LJ, Aoyama T, 2015. GLABRA2 Directly Suppresses Basic Helix-Loop-Helix Transcription Factor Genes with Diverse Functions in Root Hair Development. Plant Cell 27:2894-906. doi: 10.1105/tpc.15.00607.

Lin Y, Schiefelbein J, 2001. Embryonic control of epidermal cell patterning in the root and hypocotyl of Arabidopsis. Development 128: 3697-705. doi: 10.1242/dev.128.19.3697.

Lincoln C, Britton JH, Estelle M, 1990. Growth and development of the axr1 mutants of Arabidopsis. Plant Cell 2: 1071-80. doi: 10.1105/tpc.2.11.1071.

Marchant A, Kargul J, May ST, Muller P, Delbarre A, Perrot-Rechenmann C, Bennett MJ, 1999. AUX1 regulates root gravitropism in Arabidopsis by facilitating auxin uptake within root apical tissues. EMBO J 18: 2066-73. doi: 10.1093/emboj/18.8.2066.

Masucci JD, Schiefelbein JW, 1994. The rhd6 Mutation of *Arabidopsis thaliana* Alters Root-Hair Initiation through an Auxin- and Ethylene-Associated Process. Plant Physiol 106: 1335-1346. doi: 10.1104/pp.106.4.1335.

Menand B, Yi K, Jouannic S, Hoffmann L, Ryan E, Linstead P, Schaefer DG, Dolan L, 2007. An ancient mechanism controls the development of cells with a rooting function in land plants. Science 316: 1477-80. doi: 10.1126/science.1142618.

Shi L, Katavic V, Yu Y, Kunst L, Haughn G, 2012. Arabidopsis *glabra2* mutant seeds deficient in mucilage biosynthesis produce more oil. Plant J 69: 37-46. doi: 10.1111/j.1365-313X.2011.04768.x.

Swarup R, Friml J, Marchant A, Ljung K, Sandberg G, Palme K, Bennett M, 2001. Localization of the auxin permease AUX1 suggests two functionally distinct hormone transport pathways operate in the Arabidopsis root apex. Genes Dev 15: 2648-53. doi: 10.1101/gad.210501.

Swarup R, Kargul J, Marchant A, Zadik D, Rahman A, Mills R, Yemm A, May S, Williams L, Millner P, Tsurumi S, Moore I, Napier R, Kerr ID, Bennett MJ, 2004. Structure-function analysis of the presumptive Arabidopsis auxin permease AUX1. Plant Cell 16: 3069-83. doi: 10.1105/tpc.104.024737.

Swarup R, Kramer EM, Perry P, Knox K, Leyser HM, Haseloff J, Beemster GT, Bhalerao R, Bennett MJ, 2005. Root gravitropism requires lateral root cap and epidermal cells for transport and response to a mobile auxin signal. Nat Cell Biol 7: 1057-65. doi: 10.1038/ncb1316.

Xu W, Jia L, Baluška F, Ding G, Shi W, Ye N, Zhang J, 2012. PIN2 is required for the adaptation of Arabidopsis roots to alkaline stress by modulating proton secretion. J Exp Bot 63: 6105-14. doi: 10.1093/jxb/ers259.

Yang ZB, Geng X, He C, Zhang F, Wang R, Horst WJ, Ding Z, 2014. TAA1-regulated local auxin biosynthesis in the root-apex transition zone mediates the aluminum-induced inhibition of root growth in Arabidopsis. Plant Cell 26: 2889-904. doi: 10.1105/tpc.114.127993.

Yi K, Menand B, Bell E, Dolan L, 2010. A basic helix-loop-helix transcription factor controls cell growth and size in root hairs. Nat Genet 42: 264-7. doi: 10.1038/ng.529.

| Genes | Forward primer | Reverse primer |
| --- | --- | --- |
| *TON1A* | 5´-TGTGAGGGATGGAACAAATG-3´ | 5´-AACGCAGTTGCAAATAAAGGA-3´ |
| *EF1* | 5´-CCTTGGTGTCAAGCAGATGA-3´ | 5´-TGAAGACACCTCCTTGATGATTT-3´ |
| *PP2AA3* | 5´-TAACGTGGCCAAAATGATGC-3´ | 5´- ACCAAGCGGTTGTGGAGAAC -3 |
| *TAA1* | 5´-TGGCTAGGGACGAAGGAAGA-3´ | 5´-GCTGACTCGGACATGCTTCT-3´ |
| *YUC8* | 5´-CTCTCAGGTGCGTCAATGGA-3´ | 5´-GAGCGTTTCGTGGGTTGTTT-3´ |
| *ARF5* | 5´-GCTCGGGTTGGAAGCTTGTA-3` | 5´-ACCTTACGCATCCCACAAACT-3` |
| *ARF7* | 5´-TGCTGGAGTTCTTGGTGGTG-3` | 5´-CCACTGAGCCTCGTTTTTGC-3` |
| *ARF19* | 5´-GGCCCAATCAGACTCAACGA-3` | 5´-AGCTGTCCTTCGATGCCAAA-3` |
| *PIN3* | 5´-ATTTGGGCTCTCGTCGCTTT-3` | 5´-TGCCATTCCAAGACCAGCAT-3` |
| *GL2* | 5´-TCGGATCACTGAGACCACAA-3` | 5´-GTGTATCCCGGAACCAGTGT-3` |
| *RHD6* | 5´-GGGCGGCTTCTCCTTCT-3` | 5´-GTTTGTTTCCAGCGGATTTAG-3` |
| *RSL2* | 5´-AGTTCATTACGTCAAATTTTTGCAG-3` | 5´-TGTCCATCCCATTGAAAGCA-3` |
| *RSL4* | 5´-ACAAGACAAGAGCTTGCG-3` | 5´-ATCAGTGGCTGTCCCTTT-3` |

**Table S2. Gene-specific primer pairs used in the real-time RT-PCR experiments**.


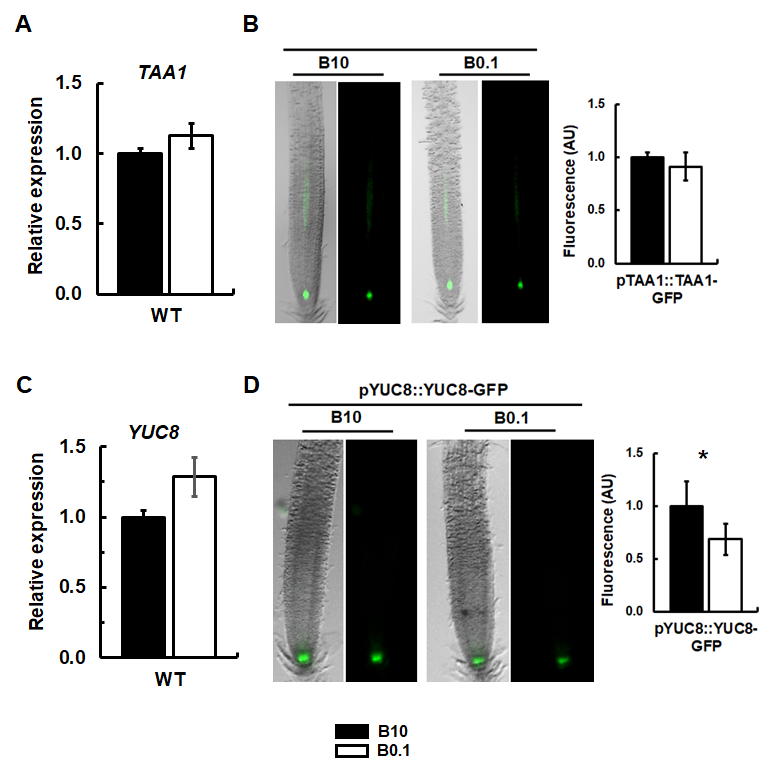


**Figure S1. Effects of B deficiency in the expression of TAA1 and YUC8 expression in the primary root tip of Arabidopsis plants.**

**(A and C)** Transcript level of *TAA1* (A) and *YUC8* (C) genes in the root tip of wild-type plants after 24 h of culture under control (10 μM B, filled bars) or deficiency (0.1 μM B, open bars) conditions. The results are given as mean ±SD (n=5 separate pools of root apices for each treatment). Asterisks indicate statistically significant differences between B treatments according to Student’s *t*-test (*p* < 0.05).

**(B and D)** Representative images (left panel) and relative GFP signal intensity (right panel) of the TAA1::GFP (B) and YUC8::GFP (D) reporter lines after 24 h of culture under control (10 μM B, B10 and filled bars) or deficiency (0.1 μM B, B0.1 and open bars) conditions. Values are given as mean ± SD of at least 10 separate plants for each B treatment. Asterisks indicate statistically significant differences between B treatments according to Student’s *t*-test (*p* < 0.05).


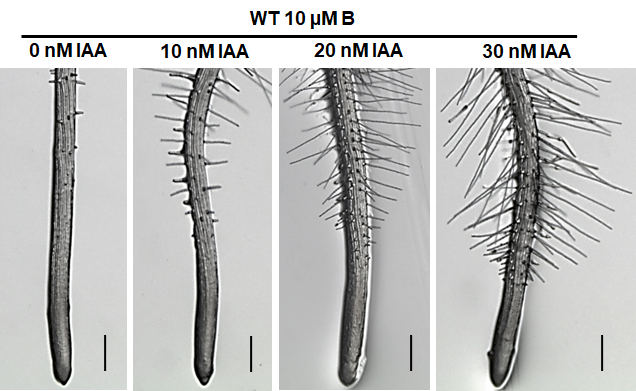


**Figure S2. Effect of auxin supply on RH development in Arabidopsis primary root tip.**

Representative images of wild-type plants grown under different IAA supplies. Six-day-old seedlings grown on 10 μM B were transferred to culture media containing either 0, 10, 20, or 30 nM IAA. RHs were imaged 24 h after transfer. Scale bars, 300 μm.


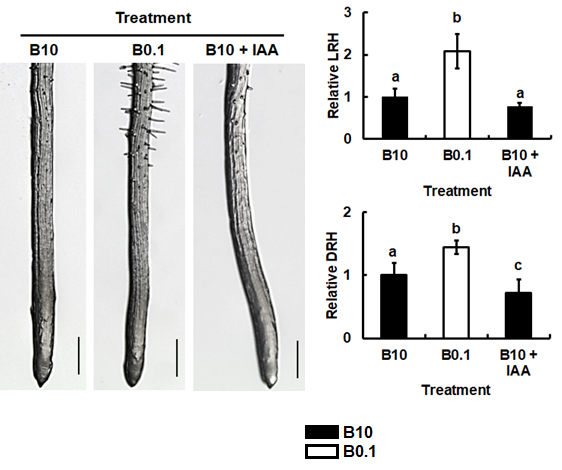


**Figure S3. The *axr1-3* mutant is insensitive to IAA supply.**

Representative images (left panel) and quantified RH length and density (right panels) of *axr1-3* mutant plants after 24 h of culture under control (10 μM B, B10 and filled bars), deficiency (0.1 μM B, B0.1 and open bars), and control plus IAA (B10 + 20 nM IAA) conditions. Values are expressed relative to control conditions (B10), which was set to 1, and given as mean ± SD of at least 12-15 separate plants for each B treatment. Different letters indicate statistically significant differences at p < 0.05 according to one-way ANOVA and post hoc Tukey test. Scale bars, 300 μm.


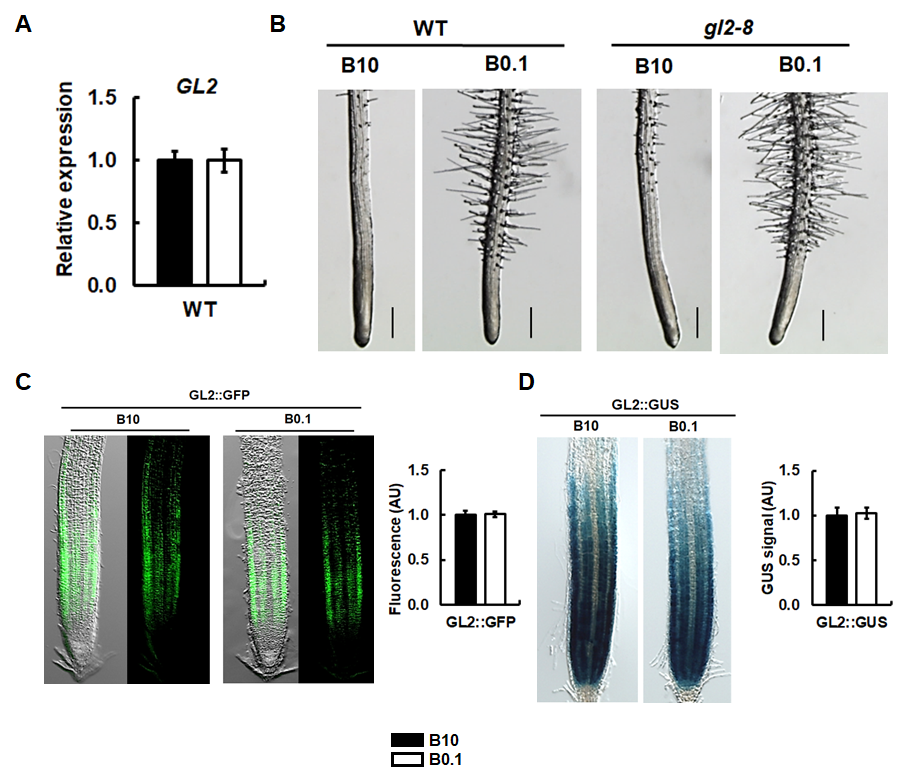


**Figure S4. Effects of B deficiency on *GL2* expression in the primary root tip of wild-type plants and RH development in *gl2-8* mutant.**

**(A)** Transcript levels of *GL2* gene in the root tip of wild-type plants after 24 h of culture under control (10 μM B, filled bars) or deficiency (0.1 μM B, open bars) conditions. The results are given as means ±SD (n=5 separate pools of root apices for each treatment). Asterisks indicate statistically significant differences between B treatments according to Student’s *t*-test (*p* < 0.05).

**(B)** Representative images of wild-type and *gl2-8* mutant plants after 24 h of culture under control (10 μM B, B10) or deficiency (0.1 μM B, B0.1) conditions. Scale bars, 300 μm.

**(C and D)** Representative images (left panel) and relative GFP/GUS signal intensity (right panel) of the GL2::GFP and GL2::GUS reporter lines after 24 h of culture under control (10 mM B, B10 and filled bars) or deficiency (0.1 mM B, B0.1 and open bars) conditions. Values are given as mean ± SD of at least 10 separate plants for each B treatment. Asterisks indicate statistically significant differences between B treatments according to Student’s *t*-test (*p* < 0.05).


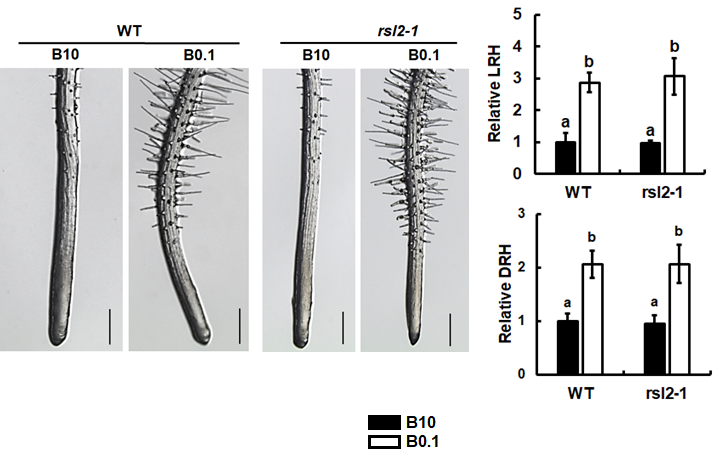


**Figure S5. The RSL2 transcription factor is not involved in the RH response to B deficiency in Arabidopsis primary root tip.**

**(A-C)** Representative images (left panel) and quantified RH length and density (right panels) of wild-type plants and *rsl2-1* mutant plants after 24 h of culture under control (10 μM B, B10 and filled bars) or deficiency (0.1 μM B, B0.1 and open bars) conditions. Values are expressed relative to those of the wild-type under control conditions, which was set to 1, and given as mean ± SD of at least 12-15 separate plants for each treatment. Different letters indicate statistically significant differences at p < 0.05 according to one-way ANOVA and post hoc Tukey test. Scale bars, 300 μm.


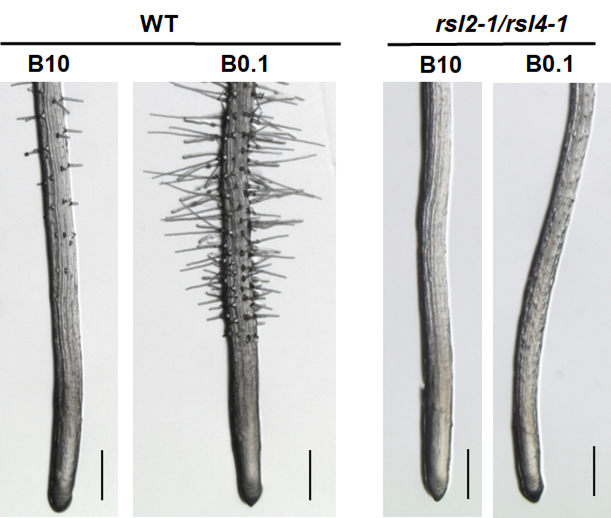


**Figure S6. The RH response to B deficiency in Arabidopsis primary root tip is inhibited in the *rsl2-1/rsl4-1* double mutant.**

Representative images of wild-type plants and *rsl2-1/rsl4-1 double* mutant plants after 24 h of culture under control (10 μM B, B10) or deficiency (0.1 μM B, B0.1) conditions. Scale bars, 300 μm.


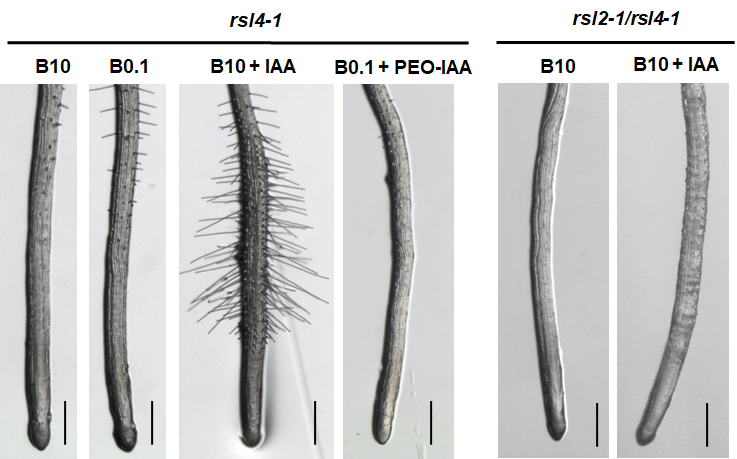


**Figure S7. RH phenotype of *rsl4-1* and *rsl2-1/rsl4-1* mutants in response to IAA or PEO-IAA supply.**

Representative images of *rsl4-1* and *rsl2-1/rsl4-1* mutant plants after 24 h of culture under the following conditions:

*rsl4-1*: control (10 μM B, B10), deficiency (0.1 μM B, B0.1), control plus 20 nM IAA (B10 + IAA), or deficiency plus 5µM PEO-IAA (B0.1 + PEO-IAA) conditions.

*rsl2-1/rsl4-1*: control (10 μM B, B10) or control plus 20 nM IAA (B10 + IAA).

Scale bars, 300 μm.
